# Thermal and chemical unfolding and refolding of a eukaryotic sodium channel

**DOI:** 10.1016/j.bbamem.2009.02.005

**Published:** 2009-06

**Authors:** Kalypso Charalambous, A.O. O'Reilly, Per A. Bullough, B.A. Wallace

**Affiliations:** a Department of Crystallography, Birkbeck College, University of London, London WC1E 7HX, UK; b Krebs Institute, Department of Molecular Biology and Biotechnology, University of Sheffield, Sheffield S10 2TN, UK

**Keywords:** CD, circular dichroism, DDM, dodecyl maltoside, TTX, tetrodotoxin, VGSC, voltage-gated sodium channel, cmc, critical micelle concentration, Voltage-gated sodium channel, Protein folding, Membrane protein, Secondary structure, Circular dichroism spectroscopy, Toxin binding

## Abstract

Voltage-gated sodium channels are dynamic membrane proteins essential for signaling in nervous and muscular systems. They undergo substantial conformational changes associated with the closed, open and inactivated states. However, little information is available regarding their conformational stability. In this study circular dichroism spectroscopy was used to investigate the changes in secondary structure accompanying chemical and thermal denaturation of detergent-solubilised sodium channels isolated from *Electrophorus electricus* electroplax. The proteins appear to be remarkably resistant to either type of treatment, with “denatured” channels, retaining significant helical secondary structure even at 77 °C or in 10% SDS. Further retention of helical secondary structure at high temperature was observed in the presence of the channel-blocking tetrodotoxin. It was possible to refold the thermally-denatured (but not chemically-denatured) channels in vitro. The correctly refolded channels were capable of undergoing the toxin-induced conformational change indicative of ligand binding. In addition, flux measurements in liposomes showed that the thermally-denatured (but not chemically-denatured) proteins were able to re-adopt native, active conformations. These studies suggest that whilst sodium channels must be sufficiently flexible to undergo major conformational changes during their functional cycle, the proteins are highly resistant to unfolding, a feature that is important for maintaining structural integrity during dynamic processes.

## 1 Introduction

Voltage-gated sodium channels (VGSCs) [1] are large membrane proteins associated with the propagation of action potentials in excitable cells. In particular they mediate the early sodium currents responsible for the rising phase of depolarisation by forming a sodium-selective pathway across membranes. VGSC mutations are associated with many pathophysiologies including cardiac arrhythmias, epilepsy and other channelopathies [2], hence underlining their importance as drug targets.

Extensive functional characterisations of these channels have been carried out [1] and although there have been a number of studies to characterise their secondary [3–6] and tertiary structures [7], and to model their 3-dimensional structures [4,8,9] the molecular basis of their functional/structural relationships is still poorly understood. VGSCs, like other membranes proteins, are notoriously challenging to study in vitro due to their low natural expression levels in vivo and the inherent difficulties associated with their overexpression and purification. As a result much work on VGSCs to date has focused on channels from the electric organ (electroplax) of the eel *Electrophorus electricus* where these channels constitute a high proportion of the membrane proteins present.

Within the VGSC family high sequence homology is observed across species and tissue-specific types. The sodium channel from the electric eel electroplax is approximately 50% identical to the human sodium channels located in the central and peripheral nervous systems, Na_V_1.1 and Na_V_1.7, respectively, 51% identical to the human heart channel (Na_V_1.5) and 59% identical to the one from human muscle (Na_V_1.4). More importantly, there is high similarity in the hydrophobicity patterns and essential residues, thus increasing the probability of the various VGSCs sharing very similar three-dimensional structures. From its cDNA sequence [10], the voltage gated sodium channel from the electric eel is predicted to consist of 1820 amino acids with a corresponding molecular weight of 208 kDa. Similar to human VGSCs, it is proposed to consist of four homologous domains (DI–IV) with each domain consisting of six transmembrane spanning segments (S1–S6), and a pore loop and extensive intracellular linking regions between each domain and extended N- and C-terminal regions. The extracellular loops between S5 and S6 are heavily modified with sugar moieties thus resulting in a glycoprotein with a total mass of ∼ 280 kDa [4].

Ion channels are dynamic molecules, adopting different conformations associated with different functional states, including closed, open and inactivated forms. Comparisons of crystal structures from the homologous potassium channel family reveal that large structural changes [11] occur between open and closed states. In addition, it has been shown by circular dichroism (CD) spectroscopy that eel sodium channels adopt different conformations in the presence of channel opening and blocking ligands [8]. Their flexibility is obviously key to their activity. Hence it is of interest to examine whether or not this flexibility leads to them being relatively stable to thermal and chemical denaturation treatments, and whether locking of their structures into a single conformational state through ligand binding can contribute further to their stability and resistance to unfolding.

This study examined the thermal and chemical unfolding of a eukaryotic VGSC and its subsequent refolding in the non-ionic detergent dodecyl maltoside (DDM). The loss and recovery of secondary structure were monitored using circular dichroism (CD) spectroscopy whilst functional activity was assayed using a fluorescence-based flux assay of protein reconstituted into liposomes.

## 2 Materials and methods

### 2.1 Materials

Monoclonal IgG anti-α-2, 8 polysialic acid was a kind gift from Prof. R. Gerardy-Schahn from the Institut fur Physiologische Chemie/Proteinstruktur, Medizinische Hochschule, Hannover, Germany. *E. coli* lipids were purchased from Avanti Polar Lipids whilst the detergent dodecyl maltoside was obtained from Anatrace. Sodium Green dye was from Invitrogen and all other materials were purchased from Sigma Aldrich at the highest purity available.

### 2.2 Isolation and solubilisation of eel membranes

Sodium channels were purified from frozen electroplax tissue following the method previously described [8], with the following modifications which increased the yield and purity of the sample: Thawed, diced electroplax tissue was suspended in 5 volumes of buffer A (50 mM NaCl, 20 mM Tris buffer pH 7.5, 5 mM EDTA, 1 mM EGTA, 0.1 mM phenylmethylsulfonyl fluoride, 0.02% NaN_3_), homogenized, filtered and centrifuged as previously described. The pellets were resuspended in buffer A at 1.5 mg/ml and stored at − 80 °C. The membranes were solubilised with 1% DDM by homogenizing the suspension followed by continuous mixing for ∼ 2 h at 4 °C. Finally, solubilised membranes were isolated by centrifugation at 100,000 ×*g* for 1 h at 4 °C to remove insoluble material.

### 2.3 Immunoaffinity chromatographic purification of sodium channels

Purified IgG was coupled to protein A sepharose (Sigma Aldrich) similar to the method previously described by Schneider et al. [12]. Briefly, ∼ 2 mg of antibody per 1 ml of wet beads was mixed for 1 h at 4 °C and subsequently washed twice with 0.2 M sodium borate pH 9 by centrifugation at 10,000 ×*g* for 30 s. Coupling was achieved by incubating the antibody/bead slurry with 20 mM dimethylpimelimidate for 30 min at room temperature. The reaction was stopped by washing with 0.2 M ethanolamine, centrifuged at 10,000 ×*g* for 30 s and incubated with 0.2 M ethanolamine for 2 h at room temperature. Finally the antibody coupled beads were resuspended in 0.02% NaN_3_, 20 mM Tris buffer, pH 7.5 and loaded into a column following washing by centrifugation 10,000 ×*g* for 30 s with 20 mM Tris buffer, pH 7.5. The IgG affinity resin was equilibrated with buffer B (buffer A and 0.1% DDM) and the solubilised membrane fraction was bound to it by continuous mixing for 2 h at 4 °C. The protein was eluted using a linear salt gradient of buffer B supplemented with 2 M KCl. The eluted protein was buffer-exchanged into buffer C (0.1% DDM, 20 mM Tris, pH 7.5) and concentrated in a concentrator with a 100 kDa cut-off membrane (Millipore). Insoluble material was removed by centrifugation at ∼ 500,000 ×*g* for 1 h at 4 °C. Protein samples were stored in buffer B, but were exchanged into sodium-free buffers prior to structural and functional studies.

### 2.4 Circular dichroism spectroscopy

CD spectra were obtained using an Aviv 215 spectropolarimeter that had been calibrated using camphor sulfonic acid for optical rotation and benzene vapor for wavelength, and fitted with a modified sample/detector geometry suitable for examining scattering samples such as membranes. A calibration plot was created of the actual temperature inside the CD sample cell (determined from a thermocouple reading) versus the set temperature. Spectra were collected at 0.5 nm intervals over the wavelength range from 280 to 190 nm in a 0.01 cm pathlength Suprasil cuvette. Measurements were only made down to wavelengths where the instrument dynode voltage indicated that the intensity of the light reaching the detector was sufficient for accurate detection [13]. Three scans were collected for each protein sample and baseline (which consisted of the column flow-through following protein concentration). Using the CDtools processing package [14], the averaged baseline spectra were subtracted from the corresponding averaged protein spectra, and the spectra smoothed with a Savitsky-Golay filter, and scaled to delta epsilon (molar circular dichroism) units using a mean residue weight value of 114.5 Da.

Secondary structure analyses were undertaken with the interactive server DICHROWEB [15], using the CONTINLL algorithm [16] and reference data set 7 [17]. This data set was chosen for this study of folded and unfolded proteins as it contains spectra of both folded and denatured proteins, and was therefore expected to be more appropriate as a reference database than ones composed of only folded proteins. Indeed it produced the best results of all the available reference databases in Dichroweb (data not shown).

The normalized root mean square (NRMSD) parameter [18] was calculated for each analysis as it is a measure of the quality of the fit of the calculated structure to the experimental data. NRMSD values < 0.1 suggest that the calculated and experimental spectra are in close agreement.

Protein concentration was determined using a Nanodrop 1000 UV spectrophotometer. The web-based Prot Param software was used to calculate the molar extinction coefficient of the protein [19].

### 2.5 Thermal denaturation/refolding studies

A thermal denaturation “melt” curve was obtained between 25 °C and 77 °C at intervals of 2 °C, measured at a constant wavelength of 222 nm (3 min equilibration after reaching each set point) using a 0.01 mm pathlength cell, with a concentration of 0.95 mg/ml.

In addition, the sodium channel was examined in the presence and absence of the ligand tetrodotoxin (TTX) (1 μM), adding 1 μl (2% of the total volume) of either buffer or buffer including TTX to ensure that the protein concentrations (0.62 mg/ml) in all samples were the same. The baseline spectra collected for the samples with TTX also contained the toxin. Spectra of the same samples were obtained at 25 °C and 77 °C. For refolding, the samples that had been heated to 77 °C were returned to 25 °C over a period of ∼ 5 min and equilibrated for a further 3 min before measurements. The equilibration times were chosen as they were sufficient to result in no further changes on repetitive measurements at the same temperature following further waiting times. A reduction in the HT was observed following cooling to 25 °C from 77 °C compared to the non-heated sample, indicating a reduction in absorption, which suggests a slight loss in material during the refolding process.

Data analysis of the “thermal melt” curves was carried out in Origin (version 6.0) using a Boltzman sigmoidal fit function.

### 2.6 Chemical denaturation/refolding studies

Chemical denaturation studies of the sodium channel were carried out in the presence of 0.02%, 0.08%, 0.1%, 1%, 5% and 10% SDS. Protein denaturation was achieved by diluting protein samples with different ratios of 10% SDS in buffer and buffer to produce a constant final protein concentration of 0.62 mg/ml in each case. Samples were incubated at 25 °C for 15 min prior to spectral measurements. CD spectra were collected at 0.5 nm intervals over the wavelength range from 280 to 190 nm at 25 °C in a 0.01 cm pathlength Suprasil cuvette. Refolding was attempted using the detergent dilution method that has been successful for refolding other helical membrane proteins [20,21]; the SDS concentration was diluted to below its cmc with 20 mM Tris buffer, pH 7.5 containing 0.1% DDM and subsequently concentrating the sample using a concentrator with a 100 kDa cut-off membrane (Millipore) to ∼ 100 μl. Protein concentration was determined using a Nanodrop 1000 UV spectrophotometer and subsequently adjusted to 0.62 mg/ml before spectra were collected following the same method as described above. To investigate whether an SDS-denatured sample was able to bind ligand, TTX was added to the sodium channels that had previously been treated with 0.1% SDS, in the manner described above for the native protein.

### 2.7 Reconstitution of channels into *E. coli* liposomes

Lipid vesicles were prepared by dissolving *E. coli* lipids in a mixture of 1:1 chloroform:methanol and freeze drying under vacuum. Lipids were then rehydrated to 20 mg/ml lipid in 1 ml of 20 mM Tris, pH 7.5 with 10 μM Sodium Green dye [22]. Liposomes with a diameter of ∼ 100 nm were formed by extrusion using a mini extruder (Avanti Polar Lipids) [23].

Sodium channel reconstitution was achieved by rapid dilution of DDM-solubilised protein into liposomes, producing a final lipid:protein ratio of 100:1 ratio (weight/weight). Initially 500 μl of 20 mg/ml liposomes was added to 100 μg of eel sodium channel (3.9 mg/ml in 0.1% DDM) and sonicated for 10 s. Subsequently DDM was diluted to below its critical micelle concentration by a 10 fold dilution of the sample with 5 ml of 20 mM Tris, pH 7.5 buffer and continuous mixing at room temperature for 20 min. Proteoliposomes were pelleted by centrifugation at 650,000 ×*g* 25 °C for 60 min. Finally, proteoliposomes were resuspended in 1 ml of Tris buffer, pH 7.5.

### 2.8 Electron microscopy

DDM-solubilised channels (100 μg/ml) were applied to carbon coated grids and stained with 1% (w/v) uranyl formate. Electron micrographs were recorded at a nominal magnification of 39,000× and an underfocus of 4000–9000 Å on a Philips CM100 electron microscope, using a Gatan Multiscan 794 charge-coupled device camera.

### 2.9 Flux assay

Fluorescence flux experiments were carried out using a FluoroMax-3 spectrofluorometer (Horiba Jobin Yvon Ltd) at 25 °C. Sodium uptake into proteoliposomes was initiated by a 33-fold dilution of liposomes containing sodium channels into 20 mM Tris, pH 7.5 buffer containing 300 mM sodium chloride. Changes in sodium green fluorescence were monitored at 540 nm (1 nm bandwidth, excitation at 512 nm) following an ∼ 10 s mixing time, after which time the increase in fluorescence had stabilised. Channel activity was assayed both in the presence and absence of 1 μM TTX or 100 μM veratridine. TTX or veratridine was added to the proteoliposome sample and incubated for 5 min at room temperature prior to the fluorescence experiments. Following the same procedure described above, control experiments were carried out either in the absence of protein (liposomes without sodium channels but still in the presence of DDM) or in the absence of sodium chloride by diluting proteoliposomes into sodium-free buffer. Each experiment was repeated 3 times with the exception of the control carried out in the presence of sodium chloride-free buffer, which was repeated twice.

### 2.10 Sequence homology and secondary structure calculations

The homology of VGSCs was calculated by aligning sequences using ClustalW [24] followed by calculation of sequence identity using GeneDoc [25]. Annotated Swiss-Prot entries on www.expasy.org provided the sequence predictions of VGSC transmembrane and extramembranous regions. Prediction of secondary structure for these regions was calculated according to the consensus of a number of secondary structure prediction algorithms [26].

## 3 Results

### 3.1 Purification and characterisation of VGSCs from electroplax tissue

**Fig. 1:**
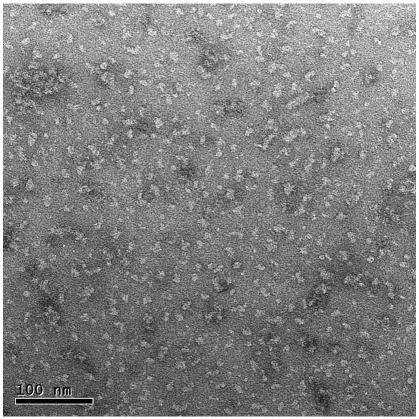
Electron micrograph of purified sodium channels from *Electrophorus electricus* electroplax: isolated sodium channels following purification in 0.1% DDM and negative staining with uranyl formate.

Membranes were initially solubilised in 1% DDM. Extended polymers of α-(2,8)-linked polysialic acid, found exclusively on VGSCs in electric eel electroplax membranes [27] enabled channels to be purified to homogeneity using a single immunoaffintiy chromatography step. In this study, mab735, a murine monoclonal IgG antibody, formed the immunoaffinity binding agent. SDS PAGE analysis revealed a single band with an apparent molecular mass of ∼ 260 kDa whose identity was further confirmed by Western blotting using anti-α-(2,8)-polysialic acid IgG (data not shown). Electron micrographs (Fig. 1) show that the preparation of purified sodium channels was relatively free of aggregates with isolated particles appearing of the order of 90 to 150 Å in diameter, depending on how they lay on the specimen support. This is in agreement with previous single particle cryo-EM studies of the protein in LUBROL-PX detergent [7].

**Fig. 2:**
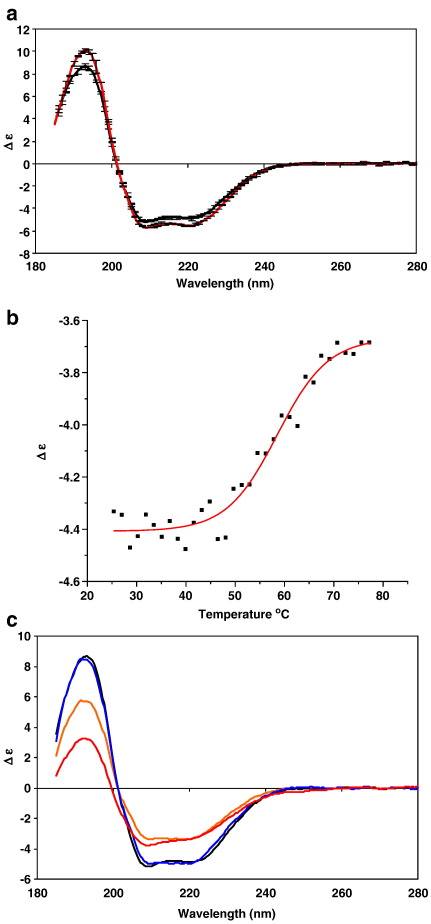
Circular dichroism spectroscopy of sodium channels in 0.1% DDM: (a) spectra in the presence (red) and absence (black) of TTX at 25 °C. Error bars represent one standard deviation in the repeated measurements. (b) Thermal denaturation curve as a function of temperature. The ellipticities were monitored at a wavelength of 222 nm over the temperature range from 25 to 77 °C. The sigmoidal fit was carried out using Origin v.6.0. (c) Spectra at 25 °C (black), at 77 °C (red), after refolding from 77 °C to 25 °C (orange) and after rescaling of the refolded sample to account for lost material (blue). All CD spectra are plotted in delta epsilon units with dimensions of M^− ^^1^ cm^− 1^ residue^− 1^.

Consistent with previous studies in other detergents [8], CD studies of the eel sodium channels showed the protein in the detergent DDM is predominantly (∼ 50%) α-helical (Table 1). DDM was chosen for this study because not only did it result in active protein (see below) but also because the detergent was of a defined chemical species with an appropriate critical micelle concentration. In detergents, the ligand-free protein corresponds to an equilibrium mixture of conformational states [8]. Circular dichroism experiments in the presence and absence of the channel-blocking toxin tetrodotoxin demonstrated that sodium channels solubilised in DDM were able to bind ligand (Fig. 2a) and undergo a conformational change to the blocked state. TTX-bound channels produced a CD spectrum of moderately larger magnitude than the channels without TTX; significantly, the spectrum for the bound state had a higher 193/222 nm ratio (1.85 compared to 1.80 for the unbound state). The higher ratio for these peaks is consistent with an increased helicity, and calculations indicate a ∼ 4% increase in helix content in the toxin-bound channel (Table 1).

### 3.2 Thermal unfolding/refolding

The thermal stability of the DDM-solubilised channels was assessed by monitoring the CD signal at 222 nm, which is primarily associated with helical structure (Fig. 2b). The initial loss in helical structure started at a temperature above 40 °C, with an apparently cooperative two-state unfolding process indicative of the absence of any significant population of unfolding intermediates. A *T*_m_ of 56 °C (*r*^2^ = 0.97) was calculated after fitting the data to a Boltzman sigmoidal function. Full spectra were then obtained on a sample that was heated to 77 °C (Fig. 2c). At 77°C, not only is there an overall decrease in the magnitudes of all peaks, but the ratio of the 193/222 nm peaks decreases from 1.80 to 0.90 corresponding to a calculated 19% reduction in helical content (Table 1). The goodness-of-fit parameter (NRMSD) was < 0.1 suggesting that the calculated secondary structures correspond well with the experimental data.

Following cooling from 77 °C to 25 °C, a further spectrum was obtained. The relative shape of the curve, i.e. the 193/222 ratio increased to a value (1.71) similar to that of the unheated sample. However, an overall decrease in spectral magnitude was observed with a concomitant decrease in the simultaneously measured high tension (HT) spectrum (effectively equivalent to absorbance) indicative of the loss of some material due to aggregation/precipitation. This kind of loss is not uncommon in refolding studies [28]. To compensate for the amount of protein lost, the refolded spectrum was rescaled to the 222 nm peak of the initial spectrum (Fig. 2c). The resulting curve was very similar to that of the unheated protein and the net secondary structure analysis of the protein remaining in solution was calculated to be 40% helix, demonstrating a considerable amount of reversible unfolding. The percentage of correctly refolded protein could be determined quantitatively using singular value deconvolution analyses [29]. Whilst it was estimated from the HT and CD scaling that approximately 1/3 of the protein was lost through aggregation/precipitation, of the protein that remained in solution roughly 60% refolded to the native conformation.

Sodium channels in their TTX-bound conformation exhibited an increased tolerance to heat denaturation compared to their unbound counterpart: The spectrum decreased to a markedly lesser extent than the 77 °C spectrum without TTX (data not shown), resulting in a smaller (16%) loss of helical structure (Table 1). After return to 25 °C, the resulting scaled structure was nearly the same as the starting structure (48% helical).

The toxin's ability to enhance stabilisation of the protein structure is further evidence of toxin binding, in addition to the observed spectral changes of the native protein at 25 °C with and without TTX.

### 3.3 Chemical (SDS) unfolding/refolding

**Fig. 3:**
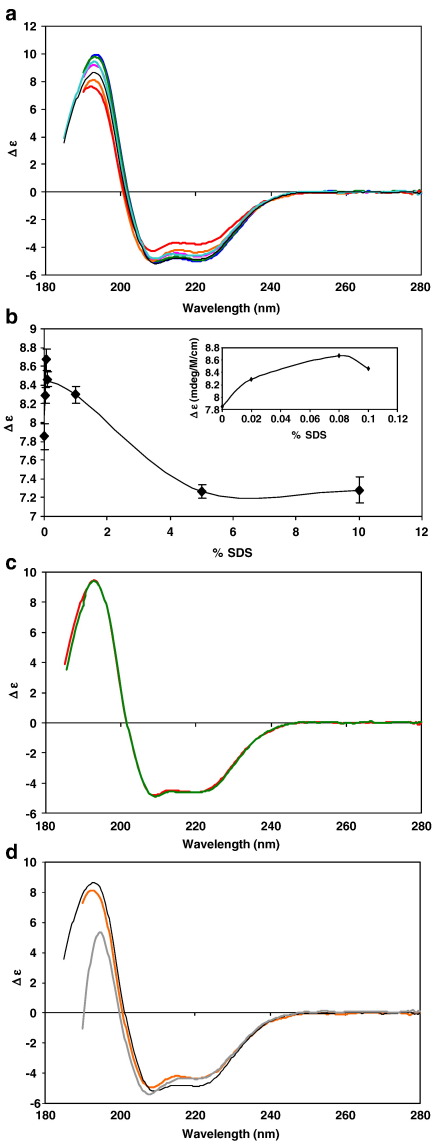
Circular dichroism spectroscopy of sodium channel showing the effect of SDS treatment on protein solubilised in 0.1% DDM: (a) channels with 0% SDS (black), 0.02 % (blue), 0.08% (cyan), 0.1% (green), 1% (pink), 5% (orange) and 10% (red) SDS, all at 25 °C. (b) SDS denaturation curve, monitored at a wavelength of 222 nm. Error bars indicate the reproducibility level for independent experiments and for clarity an inset shows a zoomed view of the low SDS concentrations region of the curve. (c) Channels with (red) and without (green) TTX added after treatment with 0.1% SDS. (d) Channels in 0% (black) and 5% SDS (orange), and after attempts to refold from 5% to 0% SDS (grey), all at 25 °C.

Increasing amounts of SDS ranging between 0.02% and 10% were used to induce protein unfolding (Fig. 3a). The helical content of the VGSC increased at the lower SDS concentrations, but as the detergent concentration increased to above the critical micelle concentration (cmc), the helical content – as monitored by the magnitude of the 222 nm peak – progressively decreased (Fig. 3b). SDS-induced unfolding does not appear to follow the simple two-state process found with heat denaturation. Instead the following phenomena were observed: a) below the cmc, the helical content increases, which we propose could result from detergent interactions with the extramembranous regions, causing them to undergo conformational rearrangement into more helical structures, b) above the cmc the displacement of the DDM detergent may promote the partial unfolding of the transmembrane regions in this relatively harsh detergent. The absence of a single isodichroic point for all the spectra (Fig. 3a) supports the suggestion that two different types of folding events occur depending on the concentration of SDS and, crucially, whether it is above or below the detergent critical micelle concentration.

Channels solubilised in 0.1% SDS produced identical spectra in the presence and absence of TTX (Fig. 3c), suggesting that the denatured channels are unable to undergo the TTX-induced conformational change seen for the native channels (Table 1). TTX binds in the selectivity filter of the channel, located at the extracellular mouth of the pore [30]. The apparent inability of TTX to induce a conformational change in chemically-denatured channels is consistent with the suggestions that the initial SDS effects involve conformational rearrangements of the extramembranous region. SDS-treated samples produced different spectra in both magnitude and shape compared to thermally-denatured samples. In addition, attempts were made to refold the protein using the detergent dilution method to minimise protein aggregation/precipitation that can result from other detergent removal methods such as potassium chloride-induced detergent precipitation. However, they were not successful in this case. The spectrum produced after the removal of the SDS (Fig. 3d) was very different from that of the native protein, but similar to that of the SDS-solubilised protein, except that the 193 nm peak was dramatically decreased. Also the NRMSD for the calculated fit for the protein “refolded” from SDS sample was very high (Table 1). These observations are consistent with the protein aggregating upon removal of the SDS. Furthermore, an increase in the HT signal was observed indicative of an increase in absorption, which could also be attributed to scattering of aggregated protein.

### 3.4 Flux activity of native, unfolded and refolded channels

**Fig. 4:**
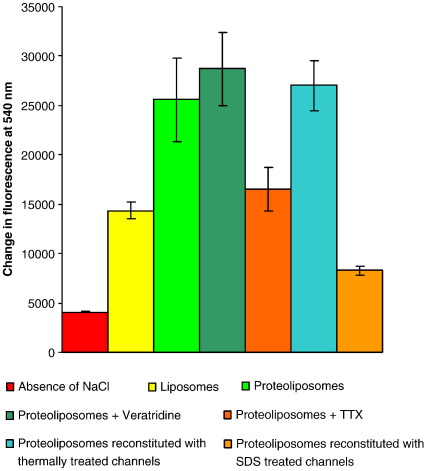
Flux assays of sodium channels: changes in Sodium Green fluorescence at 540 nm following the rapid dilution of proteoliposomes into sodium-free buffer, liposomes diluted into sodium-rich buffer, proteoliposomes diluted into sodium-rich buffer, proteoliposomes preincubated with 200 μM veratridine and diluted into sodium-rich buffer, proteoliposomes preincubated with 1 μM tetrodotoxin and then diluted into sodium-rich buffer, proteoliposomes reconstituted with protein heated to 77 °C and then cooled to 25 °C, and proteoliposomes reconstituted with protein treated with 5% SDS . Error bars indicate one standard deviation in the values of the repeated measurements.

The functional activities of the native, unfolded and “refolded” sodium channel samples were assayed following their reconstitution into liposomes using a fluorescence-based assay (Fig. 4). This assay also enables detection of functional toxin-binding capabilities of the proteins (in addition to the spectroscopically-detected toxin binding described above). Control experiments consisting of liposomes containing the channels diluted in buffer in the absence of sodium exhibited only a slight increase in fluorescence. Dilution of protein-free liposomes into sodium-rich buffer revealed that leakage was significantly less (< 50%) than the increase in fluorescence observed in liposomes containing protein, thus indicating the presence of channels enabling sodium flux. Significantly, channel-induced flux was shown to be modulated by the addition of VGSC neurotoxins, thus demonstrating that reconstituted channels were active and able to bind ligands. A small increase in sodium flux was observed in proteoliposomes preincubated with veratridine, a sodium channel-specific drug that generates steady-state activation. The small effect is likely because under the conditions of reconstitution, many of the channels are already in an open state. In contrast proteoliposomes pre-treated with the channel blocker TTX showed a marked decrease in fluorescence, mirroring fluorescence levels seen in control experiments in the absence of sodium channels. Again, this large effect is likely a consequence of many of the proteins in the untreated samples being in an initial open state.

“Refolded” proteins were also reconstituted into liposomes and their flux properties examined (Fig. 4). Proteoliposome samples consisting of protein that had been heated to 77 °C and then returned to 25 °C behaved in a similar manner to proteoliposome samples reconstituted with native DDM-solubilised channels at room temperature. This suggests that the unfolding process induced through thermal denaturation is largely reversible. However, this assay is unable to establish the proportion of proteins refolded correctly. In contrast, SDS denatured proteins did not appear to refold into functional channels following detergent exchange into DDM, as their flux levels mirrored those of control samples (liposomes without any channels in them). It thus appears that the reconstituted protein is not functional and that the SDS unfolding undertaken in this study was not a reversible process.

## 4 Discussion

Studies on unfolding and refolding of proteins and the parameters involved in these processes are important for gaining insight into their stability and activity and can inform efforts to produce correctly folded protein for functional and structural characterisation [31]. In nature, complex pathways operate to prevent the accumulation of misfolded proteins. However, protein misfolding occurs and has been implicated in diseases including cystic fibrosis, Alzheimer's and Parkinson's; their treatments challenge modern medicine. Frequently pathologies linked to misfolded proteins are associated with mutations but studies have shown that environmental factors such as temperature fluctuations, protein overexpession and oxidative stress can also result in protein misfolding. In vivo membrane proteins reside in the dynamic lipid bilayer, which is both anisotropic and physically restrictive. They have evolved to survive and function in this environment, the complexity of which represents a major obstacle limiting study of this protein class. Consequently our knowledge of membrane protein folding and refolding mechanisms is less advanced than that of soluble proteins. Unique factors involved in membrane protein folding include the anisotropic and amphipathic nature of the lipid bilayers and detergent micelles necessary for maintaining membrane protein structure and solubility, and the different susceptibilities of the extramembranous and transmembranous domains to denaturation [32,33]. This study has examined the nature and factors involved in the thermal and chemical unfolding and refolding of the VGSC from electric eel.

The eel VGSC is a single chain, primarily helical, polytopic transmembrane protein. Its CD spectrum suggests that in DDM 48% of the protein is helical. Single particle electron microscopic analysis had suggested that ∼ 75% of the eel sodium channel volume is located in the cytoplasm or periplasm with only ∼ 25% forming the transmembrane portion of the protein [7]. Based on the transmembrane segments predicted from the amino acid sequence [34], the transmembrane helical content would account for only 22% of the total amino acids, which is in accord with the single particle studies. Hence, a considerable portion of the extramembranous regions must be helical to account for the total helical content. This is realistic based on calculations of helical propensity [34] for the extended loops, interdomain connectors and ends, which are calculated to be ∼ 32% helical.

VGSCs appear to be highly flexible molecules. The homologous potassium channels have been shown to undergo very significant conformational changes between their open and closed states [35–37], and thus sodium channels are expected to undergo similar flexing between functional states. This is supported by spectroscopic studies that show they undergo significant conformational changes upon binding ligands such as the drug lamotrigine and the toxin batrachotoxin [8] which shift the equilibrium balances towards inactive and open states, respectively, and TTX (this study) which blocks the pore. These different ligands have distinct binding sites in the channel conductance pathway and cause qualitatively different conformational changes. Whereas local anaesthetics and the toxins batrachotoxin and veratradine bind in the transmembrane portion of the VGSC pore, TTX binds to its extracellular mouth. TTX forms interactions with all four domains and residues involved in binding include residues of the DEKA motif, which constitutes the selectivity filter [38]. Our limited knowledge of this region of the channel (structures of potassium channel are by necessity different in this region) precludes identifying the source of increased helicity observed upon TTX binding in the present study, but it is likely to involve the extracellular regions rather than the transmembrane domains.

In this study, the thermal stability of the eel VGSC was investigated, and as with other membrane proteins [39–41], the channel was found to be relatively resistant to thermal denaturation, with a high *T*_m_, and a highly helical “denatured” state [20]. This is by comparison to thermally-denatured soluble proteins, which often have *T*_m_s closer to physiological temperature and for which the unfolded state retains much less ordered secondary structural content. This may, in part, be because the transmembrane regions, being helical, tend to be more stable than other types of secondary structures, and/or because the surrounding detergent micelle tends to restrain the membrane protein and protect it from unfolding. Although this study cannot define where the remaining helical regions are, it is tempting to speculate that since the fully “denatured” protein still has sufficient helix to account for all the transmembrane helices, it is indeed these regions, surrounded by detergent molecules, that retain their secondary structure and that analogous to soluble proteins, the extramembranous regions that are not restrained are more susceptible to unfolding. It may also help explain why the thermally-denatured protein is still capable of refolding to a native structure that is functional: the transmembrane core that forms the pore structure remains intact. TTX binding results in a further stabilisation of the protein. This stability may arise from the binding contacts TTX forms between the four domains at the pore's extracellular mouth.

For consistency, all the functional and structural experiments reported in this study were done in buffers containing low sodium, as this was a necessary condition for the flux measurements. However comparisons of the CD spectra of the protein in high salt ([8] and unpublished results) suggest that the protein may have an even slightly more helical structure under those conditions, in line with studies on other ion channels that have shown that the presence of permeating or blocking ions may act to order and stabilise channels [42–44]. Nevertheless, high thermal stability was observed even under the present conditions.

Although these observations of thermal stability but functional flexibility may seem contradictory, they can be rationalised as follows: increasing the temperature normally increases the thermal motions of proteins to the point of unfolding. However, proteins with great conformational flexibility intrinsic to their activity require greater tolerance to dynamic changes, and so may display increased resistance to unfolding by thermal stress.

The mechanism for chemical unfolding of the eel channel appears to be very different from that of thermal unfolding. SDS is a negatively-charged detergent that binds to both soluble and membrane proteins, often causing an increase in helical content at concentrations below its cmc. We postulate that at low concentrations it binds to the extramembranous regions of the VGSC, producing an increase in helicity. At concentrations above their critical micelle concentration, detergents form micellar structures, which surround the hydrophobic regions of a membrane protein. SDS and DDM detergents share the same type of hydrocarbon “tail” but have different “head groups” which may result in different interactions with proteins. The increasing amounts of SDS that were added during the chemical denaturation studies described in this paper are predicted to result in this detergent displacing DDM from the micelle, retaining solubility of protein molecules but also leading to denaturation. Reversing the process by removing SDS through lowering its concentration below the cmc in the presence of DDM did not rescue functional protein, and by inference did not lead to protein refolding to an active state. When the SDS is removed, the protein aggregates as the negative charges on the detergent that resulted in mutual repulsion of the denatured proteins are not present on DDM. SDS interferes with TTX binding even at concentrations below the cmc, so the “denaturation” in this region may occur even before the protein undergoes substantial global unfolding.

In summary, in this study we have examined the unfolding and refolding of the eel VGSC by both thermal and chemical means, and found that whilst both result in significant loss of helical structure, the thermally-unfolded proteins are capable of refolding to active forms, whilst the chemically-unfolded proteins do not refold. We postulate that one difference between these unfolding processes could be that they differentially involve the transmembrane and extramembrane regions of the molecule.

The biological relevance of these studies is with respect to the dynamics of channels: their requirement of flexibility for ion channel activity means that they must not unfold easily when subjected to conformational stress. Hence, the stability (i.e. inability to unfold) demonstrated here indicates that although these molecules can undergo significant conformational changes (effectively the ability to undergo local refolding) associated with the functioning of the channel, they do not easily undergo global unfolding, thereby maintaining the integrity of the protein during the normal functioning cycle.

## Figures and Tables

**Table 1:** Calculated secondary structures

Sample	% Secondary structure				
	Helix	Sheet	Turn	Other	NRMSD
25 °C	48	5	16	32	0.042
77 °C	29	14	17	40	0.050
77→25 (scaled)	41	15	20	25	0.029
TTX 25 °C	52	4	18	26	0.035
TTX 77 °C	36	12	18	34	0.054
TTX 77→25 (scaled)	48	13	20	20	0.022
0.02% SDS	54	5	18	24	0.045
0.08% SDS	51	9	16	24	0.045
0.1% SDS	47	11	16	26	0.051
TTX 0.1% SDS	47	11	16	28	0.010
1% SDS	49	4	16	31	0.042
5% SDS	47	3	15	35	0.040
10% SDS	45	9	15	32	0.043
5% SDS→0% SDS	56	6	20	19	0.148

NRMSD is a goodness-of-fit parameter, with low values indicative of close correspondence between calculated secondary structure and experimental data.
